# Waveform load analysis for fatigue in the printed PLA

**DOI:** 10.1016/j.heliyon.2023.e18480

**Published:** 2023-07-25

**Authors:** Moises Jimenez-Martinez, Julio Varela-Soriano, José Jorge Rojas Carreón, Sergio G. Torres-Cedillo

**Affiliations:** a Tecnologico de Monterrey, Escuela de Ingeniería y Ciencias, Via Atlixcayotl 5718, Col. Reserva Territorial Atlixcayotl, C.P. 72453 Pue, Puebla, Mexico; b SEPI-ESIME Ticoman IPN, Av. Ticomán 600, La Purísima Ticoman, Gustavo A. Madero, 07340 Ciudad de México, Mexico

**Keywords:** Additive manufacturing, Fused filament fabrication, Load waveforms, Cyclic loads, PLA

## Abstract

Additive manufacturing is fast becoming a key process to manufacture a customized design with complex geometry and one process usually employed is based on the fused filament fabrication. Up to now this method is typically employed for rapid prototyping, it is therefore their mechanical strength is lower than the components manufactured using conventional casting process. It is well known that most failures are happened under repeated loads; therefore, a functional component mandatory needs to reach endurance strength under cyclic loads. Hence, this study set out to clarify several aspects of filament fused test specimens to determine their effect on accumulated damage to then predict component life under repeated loads. In this study is considered three waveforms such as sinusoidal, triangular and square, where it is observed that the square waveform provides the most severe loads. This study therefore makes a major contribution to research on the fatigue properties of parts manufactured using fused filament by reporting their fatigue behaviour under different fatigue load conditions. It would give a better understanding to improve the mechanical prediction of PLA, thereby it might be used to manufacture a functional component instead of only a prototype or spare part.

## 1 Introduction

**Figure 1:**
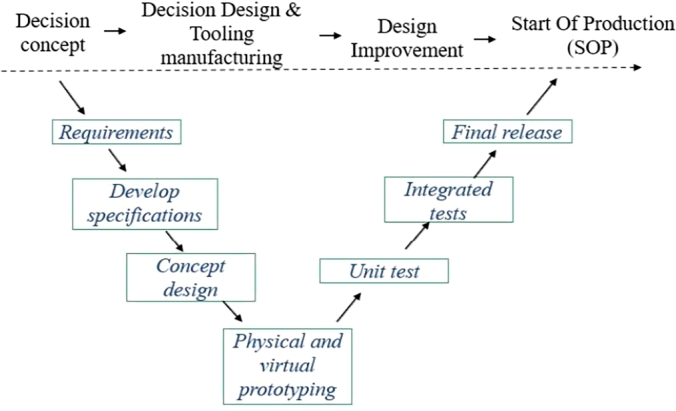
Product Evolution Process.

In the new global economy, additive manufacturing has become a central issue for manufacturing. Manufactured components must meet requirements according to the sector where they are used (energy, mobility and biomedical) [1]. These are developed by interdisciplinary teams to include different functions and process production, including chemical composition, hardness, mechanical properties under quasistatic loads and behaviour under cyclic loads [2]. It is well known that the standards can change and also can be critical according with the number of components produced and their functions [3]. High-volume production components are evaluated prior to production at different stages, as shown in Fig. 1. The tests range from the selected material, the component as a unit, the same integrated unit to a subsystem and final assembly. Additionally, the stages of development of tools are also considered, and the tests can be physical or virtual [4]. After the series of tests and optimization cycles, the final release and the start of production are carried out. In the new global economy, additive manufacturing has become a central issue for manufacturing. Manufactured components must meet requirements according to the sector where they are used (energy, mobility and biomedical) [1]. These are developed by interdisciplinary teams to include different functions and process production, including chemical composition, hardness, mechanical properties under quasistatic loads and behaviour under cyclic loads [2]. It is well known that the standards can change and also can be critical according with the number of components produced and their functions [3]. High-volume production components are evaluated prior to production at different stages, as shown in Fig. 1. The tests range from the selected material, the component as a unit, the same integrated unit to a subsystem and final assembly. Additionally, the stages of development of tools are also considered, and the tests can be physical or virtual [4]. After the series of tests and optimization cycles, the final release and the start of production are carried out.

Although standard production processes such as stamping, foundry, moulding and substracting are the major manufacturing high-volume processes, their behaviour under dynamic loads is still being researched. Different methodologies such as systems engineering are implemented in original equipment manufacturers to integrate the whole product lifecycle [5]. Virtual simulation is used to understand and improve the final behaviour of the product as well as the manufacturing process. The manufacturing process has an effect on the mechanical performance of components, regardless of the additive or subtractive manufacturing process employed [6].

**Figure 2:**
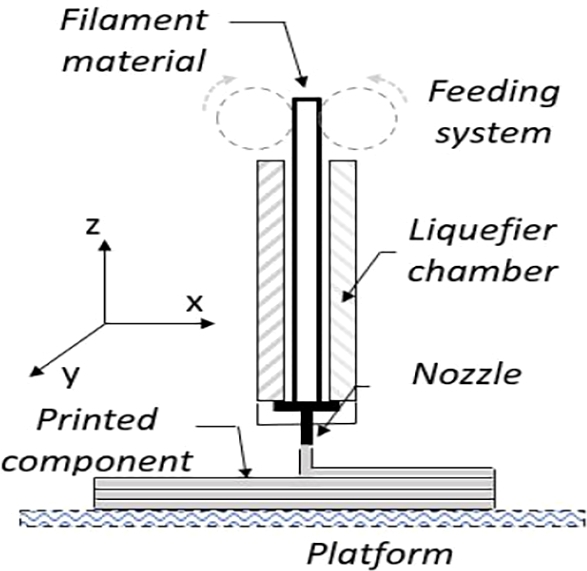
Schematic representation of the printing process.

Subtractive manufacturing starts with a solid component that is shaped by removing materials with different processes such as drilling, cutting and grinding using manual processes or processes with toolpaths. These processes can generate stress concentrators directly by subtracting material or indirectly by the process itself, for example, heating [7]. The structural strength of the component depends on the material, design, material properties, mechanical loads and manufacturing process, and the stresses or design changes during the manufacturing process modify the component strength because they generate a prestress initial condition due to the remelting process during cooling [8]. This printing process can be described as follows, there is a temperature differential when the filament flows through the nozzle to the cooling point. This difference in temperature also appears when printing layer is placed over a hot bed during. Those printing process can be considered as remelting process during cooling. It can be also the origin to have a different mechanical behaviour in the direction perpendicular to the printing bed [9]. Additive manufacturing (AM) is the method of forming components by adding material. In fused deposition modelling, the material is solidified after melting on the nozzle to obtain the geometry set by its movement (Fig. 2). This process generates complex designs and process conditions, nevertheless it has the potential for engineering applications such as mobility, mechanical, biomedical, acoustic insulators and appliance industries [10]. However, there is a necessity to achieve operational requirements such as fatigue strength [11].

Extensive research has shown that Polylactic acid *PLA* is not recommended for long-term use [12]. To overcome this constraint, it is necessary to understand its mechanical response under cyclic loads, which may change the tendency to use this type of printing from temporal use as a prototype or spare part to a functional component because polymers are sensitive to the heat generated by their manufacturing process [13]. To improve the mechanical performance of components printed with PLA, have been proposed to add a constituent material, Sharifabad et al., [14] proposed to add nanocomposites to improve the mechanical performance.

Polyactide or poly(lactic acid) substitutes petroleum-based polymers. Thermoplastic filaments of PLA can be produced by chemical reaction using a fermentation of sugars. Thermomechanical degradation of printed *PLA* is developed by unfolded polymerization, it develops a failure process generating partition on the polymer chains. The accumulated damage involves hydrolysis, oxidative degradation, and intramolecular transesterification. There are cohesion problems in the printed component with thermoplastic material due to the difference in cohesion forces between printed layers and the bonding forces generated between the polymer filament after printing, resulting in a difference in the stiffness of the component. [15].

Due to their viscoelastic behaviour, polymers can be damaged by cyclic loads, even at low loads. The process of damage generated is the nucleation of the failure generating a fissure, which grows until the component fails by a crack. Mechanical fatigue generates heat and sometimes softening of the material, from the nucleation of the damage, during its propagation and until its final failure [16]. Thermoplastic materials have a high damping due to the motion that exists in the polymerization chains, however by increasing the loading speeds, such as the frequency and the deformation ratio. Additional heating is generated on the component, which accelerates the process of thermal fatigue and damage [17]. This study aims to contribute to this growing area of AM research by exploring the effect of the waveform of the load, considering that the wave has an effect on the durability of the component printed and its predicted operational life. It is also considering the peak value of a load cycle to estimate the component's durability under dynamic loads conditions. However, fatigue failure is a result of a process of accumulated damage, this involves damage accumulation over each cycle. Therefore, if the wave profile is different the damage suffered by a component would be more severe. Hence, this investigation makes important contributions by considering three different wave forms, which are analyzed to improve fatigue life prediction.

## 2 Mechanical fatigue

Accumulated fatigue damage in polymers is complex due to the dependency on temperature. During repeated loads, the accumulated damage depends on factors such as the frequency, time at level and waveform used for the load [18]. Many recent studies have shown that *PLA*-printed components can be considered isotropic with 100% infill density for repeated loads. Minor density variations such as notches act as stress concentrators [19].

Fatigue strength depends on the accumulated damage as a function of the Ultimate Tensile Strength (*UTS*) and the material mechanical properties ($C_{m}$), as expressed in Equation (1):

(1) $$S_{fe}=UTS\times C_{m}$$

**Figure 3:**
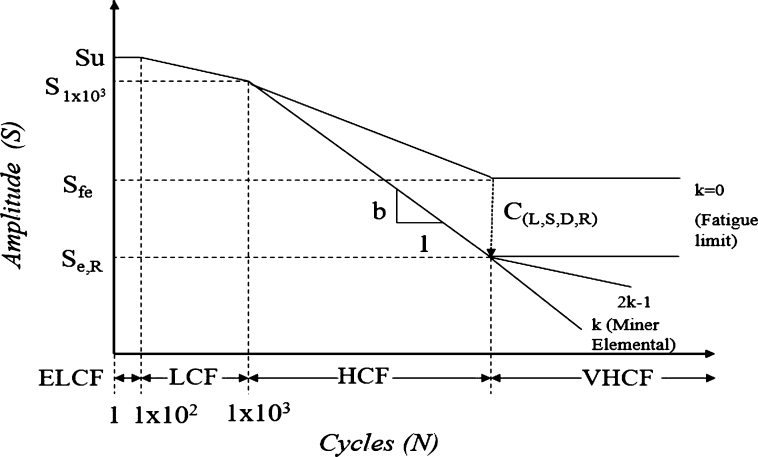
Schematic SN curve.

During fatigue assessment it is necessary to consider different factors, these reduce the fatigue strength $S_{fe}$. It is expressed by Equation (2) and Fig. 3.

(2) $$S_{e,R}=S_{fe}\times C_{L}\times C_{S}\times C_{D}\times C_{R}$$

where the load factor $C_{L}$ depends on the load characteristics (bending, torsional pure axial) and its severity. The surface finishing factor ($C_{S}$) affects delay with compressive residual stresses or advance failure nucleation as stress concentrators. The size factor $C_{D}$ represents stress concentrators as a function of geometry. The Reliability factor ($C_{R}$) included the variations on material and manufacturing process on components included in the same lot of production. A schematic curve of fatigue life is shown in Fig. 3. These curves are known as Wöhler curves or SN curve, where it is analyzed how many cycles (N) a component or material supports at a given level of Load (S). This curve is defined by an Extremely Low Cycle Fatigue (ELCF), a very Low Cycle Fatigue (LCF), the area of interest for PLA printed components is the High Cycle Fatigue (HCF), and where there is a theoretical limit of fatigue resistance, the very high cycle Fatigue (VHCF).

## 3 Experimental test

To evaluate the mechanical behaviour, many specimens were printed with the dog-bone geometry according ASTM D7791 [20]. Three different thicknesses (t) were printed at 5, 8 and 11 mm, and two widths (w) were printed at 20 and 30 mm. In observational studies, there is a potential for bias from the printing process itself for the different thicknesses used, due to the material stiffness decreasing with an increase in the thickness of the specimens [21].

The test pieces were fabricated on a commercial 3D printing machine provided by Ultra-maker and Ender, employing a *PLA* filament of red colour from the Brand colour plus 3D with a diameter of 1.75 mm. The process was carried out at a temperature of 200 °C for the liquefier chamber and 55 °C for the build platform [22], with a raster angle of 45° [23], based on that at 45°/-45° the durability is four times that when printing at 0/90° [24]. The infill density was also defined with 100% to avoid internal stress concentrators. The samples were maintained under standard laboratory conditions (room temperature) for 48 hours before to test them [25]. Other physical and mechanical properties of *PLA* are $1.24\text{ g}/{\text{cm}}^{3}$ density, 22–49 MPa UTS (Ultimate Tensile Strength) and Youngś Modulus E = 3.25 GPa. Fifty-four components were tested, 27 for each width, 9 components for each triangular, square and sine waveform, respectively.

**Figure 4:**
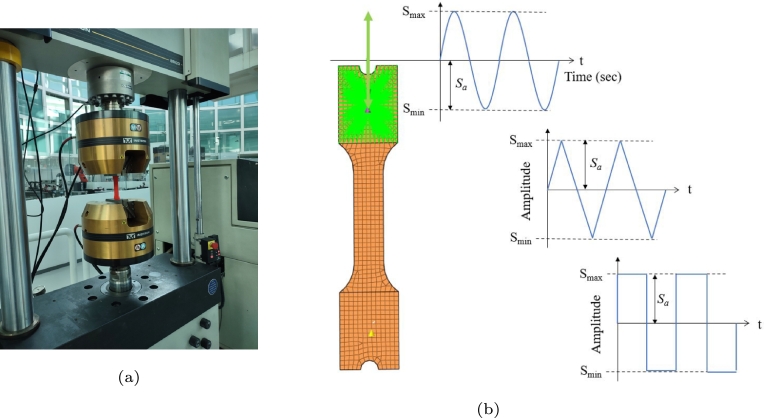
Fatigue test set up (a) test bench and (b) load profile.

An Instron uniaxial fatigue test (Instron) was employed for the fatigue test. To prevent an increasing temperature due to test heating, the frequency was 2 Hz, and the relationship between the positive and negative loads was R=-1, as shown in Fig. 4.

The stress *σ* at any moment is expressed as:

(3) $$\sigma =\sigma_{o}sin\omega t$$

where *ω* is the angular velocity of load.

For a viscoelastic material the sinusoidal stress is a variation of the strain (*ε*) with the stress including a phase lag (*δ*) as is shown in

(4) $$\varepsilon =\varepsilon_{o}sin\left(\omega t-\delta \right)$$

By combining Eq. (3) and Eq. (4), we can obtain Eq. (5):

(5) $$\sigma =\sigma_{o}sin\omega tcos\delta +\sigma_{o}cos\omega tsin\delta$$

The stiffness behaviour of the material leads to a complex modulus *E*⁎, as shown in Eq. (6).

(6) $$E⁎=\frac{\sigma }{\varepsilon }=\sqrt{E_{1}^{2}+E_{2}^{2}}$$

where $E_{1}=\left(\sigma_{o}cos\delta \right)/\varepsilon_{o}$ is in phase with the strain, and $E_{2}\left(\sigma_{o}sin\delta \right)/\varepsilon_{o}$ is out of phase.

The complex modulus can be expressed by Eq. (7):

(7) $$E^{⁎}=E_{1}+iE_{2}$$

where $i=\sqrt{-1}$, $E_{1}$ us the real modulus or storage modulus and $E_{2}$ is the imaginary modulus or loss modulus.

## 4 Finite element simulation

Data were collected from the finite element simulation to understand the mechanical behaviour of the specimen under test, and three different load profiles were evaluated for Sa=+/- 2000 N. The finite element analysis was performed using Altair program, hypermesh for the preprocessing, Radioss solver and hyperview for the postprocessing. The finite element model is generated with 668 quadrangular elements, 20 triangular elements of first order. The boundary conditions are a spatial constraint with 6 DOF=0, applied directly in the elements of one side of the component, and the load profile (Fig. 4b) in the other side using a rigid element. The mechanical properties used are: Young's modulus *E* = 3.25 GPa, a Poisson coefficient *ν* = 0.32 and a density *ρ* = $1.25e^{-9}$ $\text{tonne}/{\text{mm}}^{3}$, the failure criteria used is Von Mises [26], [27].

## 5 Results and discussion

**Figure 5:**
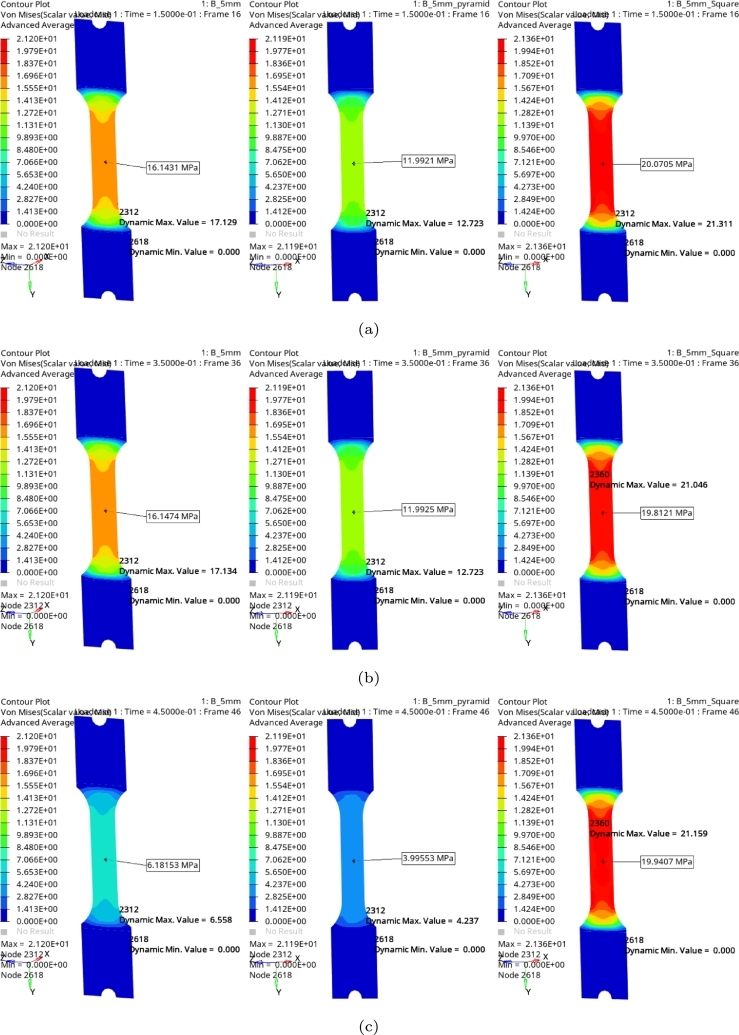
Stress results with different load profiles. Each subfigure has a sinusoidal load at the left, triangular at the middle and square load at the right: (a) 0.15 sec, (b) 0.35 sec, and (c) 0.45 sec.

Fig. 5 provides the results obtained from the simulations for three waveforms at each time. These results were extracted at three separate times, in Fig. 5a presents the results at 0.15 seconds, Fig. 5b at 0.35 seconds and Fig. 5c at 0.45 seconds. These results were obtained from the waveforms illustrated in Fig. 4a, then Fig. 5 is structured as follows: in left column provides results obtained from sinusoidal waveform, in central column presents results from triangular waveform and in right column results from square waveform, respectively. The results obtained from these simulations are summarized in Table 1. What is interesting about the data in this table is that there is a noticeably effect at each waveform, where the most critical is square shape resulting four from six charge levels have lowest level. It can be contrastive with the triangular shape, where only two cases achieve the lowest values. However, it could be expected from triangular shape due to the history of stresses during the load cycle. It also might be stated that this phenomenon is related to the change in direction of the charge unlike sine waveform case where it was observed smooth transition.

**Table 1:** Stress results at different times for a component of 5 mm thickness.

Waveform						
Time	Sinus		Triangular		Square	
	Measure	Maximum	Measure	Maximum	Measure	Maximum
0.01	1.264	1.342	0.805	0.855	14.483	15.375
0.05	6.178	6.554	4.004	4.247	19.81	21.034
0.1	11.741	12.457	8.001	8.448	20.01	21.234
0.15	16.143	17.129	11.992	12.723	20.07	21.311
0.2	18.971	20.132	15.975	16.952	19.797	21.029
0.25	19.975	21.199	19.965	21.187	20.071	21.307
0.3	18.967	20.127	15.966	16.975	20.008	21.241
0.35	16.147	17.134	11.922	12.723	19.812	21.046
0.4	11.745	12.462	7.99	8.476	20.115	21.346
0.45	6.181	6.558	3.995	4.237	19.94	21.159
0.5	0.005	0.006	0.008	0.01	0.254	0.282

**Figure 6:**
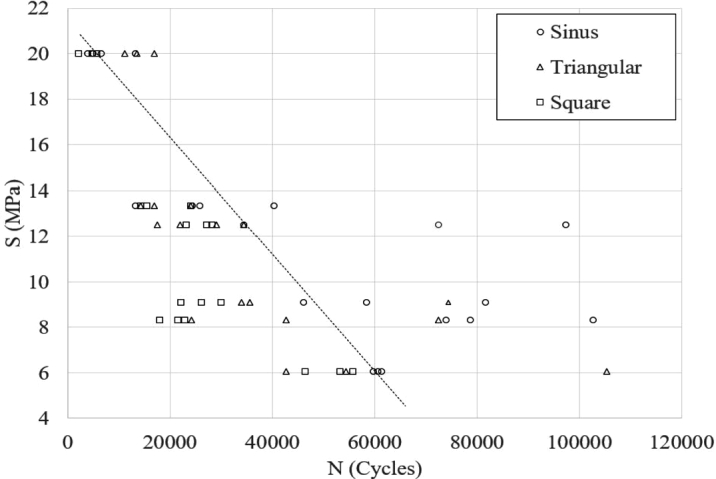
SN curve for the experimental results at different load levels.

In this study the component life is expressed using number of cycles at the failure appeared, or if there is a displacement less or grater than 0.5 mm. However, the obtained results are counterintuitive (as can be seen in Table 1) due to it was expected that the triangular waveform has less damage per cycle, resulting in a major durability at all stress levels. It is also noted that this tendency is only seen at 20 MPa of amplitude, and at 6.1 MPa of load, it has a result with the best durability at 105,431 cycles but also the worst at 42,581 cycles, as described in Fig. 6. For the remain stress values in noted that the major durability is found under a sinusoidal load. It is therefore using the linear damage rule, it can be employed the SN curve to perform the durability assessment.

Turning now to Table 2, this table provides the mean values results obtained from different experimental sets, it can be observed that the higher fatigue resistance is presented with sine waveform at 8.3 and 13.3 MPa, respectively. Further analysis shows that the median result values at 20 and 6.1 MPa are major with triangular waveforms and not with sinusoidal waveforms; it was employed thicknesses of 11 and 5 mm, respectively. It is therefore suggested that the thickness is not a factor influenced by the wave forms.

**Table 2:** Mean values for the experimental fatigue tests.

Waveform	Stress (MPa)					
	20.0	13.3	12.5	9.1	8.3	6.1
Sinus	7,130	25,896	68,116	62,036	85,138	60,537
Triangular	13,895	12,593	25,787	47,994	46,450	67,448
Square	4,188	17,954	26,202	26,088	20,797	51,719

**Figure 7:**
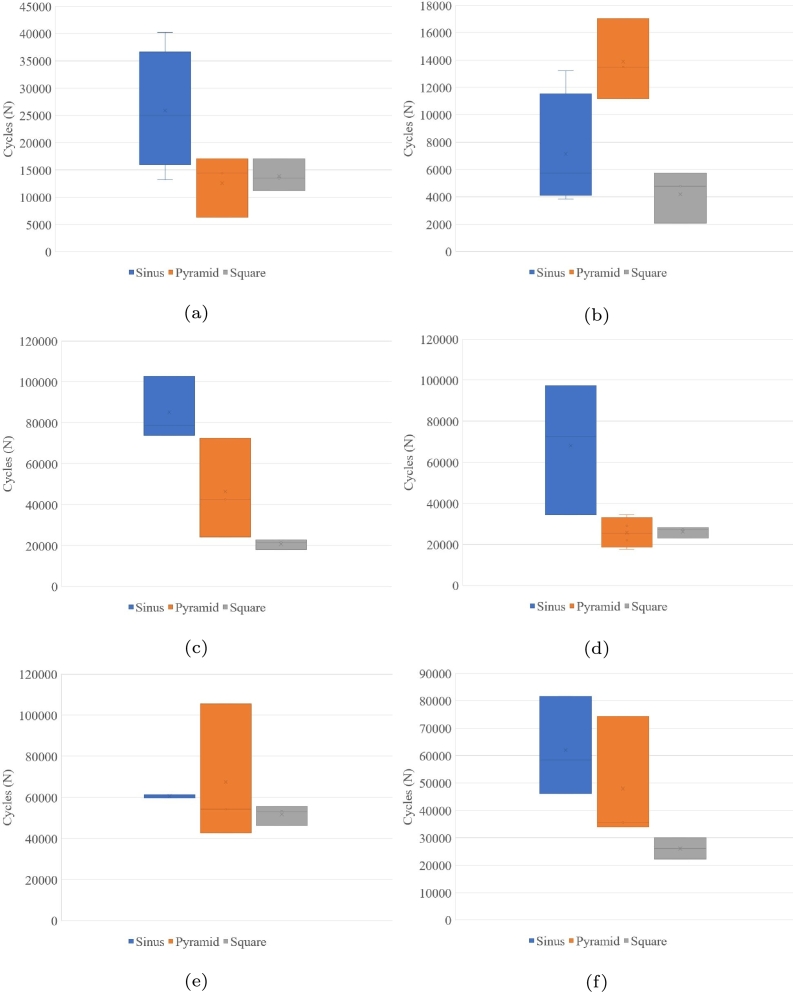
Comparative waveform experimental test, (a)design A-5 mm, (b)design B- 5 mm, (c)design-A 8 mm, (d)design-B 8 mm, (e)design A- 11 mm and (f)design-B 11 mm of thickness.

In Fig. 7 and Table 3 is summarized the statistics for a scatter analysis from the experimental results. The results obtained from simulations with thickness 5 mm are shown in Figs. 7a and 7b. It can be seen from these figures that the square load generates results like the more aggressive waveform. From these two figures, it is also observed that square form has a similar harmful performance and better performance with sinusoidal waveform load. Likewise, in Figs. 7(c-d) has also the same tendency between the sinusoidal and triangular loads with a thickness of 8 mm, and the main difference is the scatter. In contrast, the triangular load does not have the same tendency over all Fig. 7(a-d). The more surprising correlation is with the triangular waveform in Figs. 7e and 7f.

**Table 3:** Standard deviation per load.

Design	Waveform		
	Sinus	Triangular	Square
A-5 mm	0.198	0.23	0.122
B-5 mm	0.231	0.091	0.236
A-8 mm	0.075	0.237	0.053
B-8 mm	0.231	0.129	0.045
A-11 mm	0.005	0.203	0.041
B-11 mm	0.124	0.19	0.065
Mean-A	0.093	0.224	0.072
Mean-B	0.196	0.137	0.115

The findings reported here suggest that it seems that it is not possible to define a factor to estimate the SN curve for PLA among sinusoidal, triangular and square waveforms; however, among the sinusoidal and square waveforms, as well as triangular and square waveforms, the most severe of the combinations is the square waveform load between 53% and 75% due to a greater energy dissipation per cyclic load.

**Figure 8:**
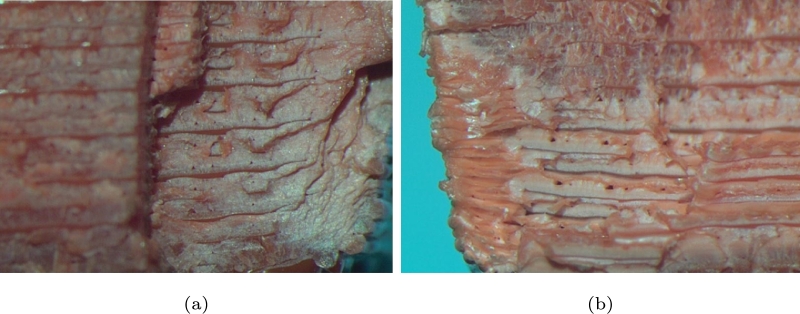
Internal failure due to the sinusoidal waveform at 13,206 cycles: (a) crack direction and (b) chevron pattern.

**Figure 9:**
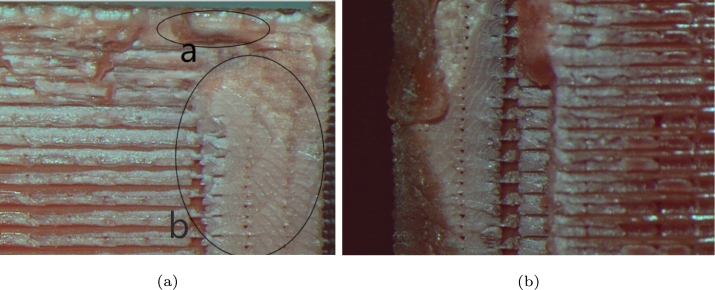
Internal failures due to the triangular waveform at 13,331 cycles, (a)rubbed surface and (b) beach marks.

**Figure 10:**
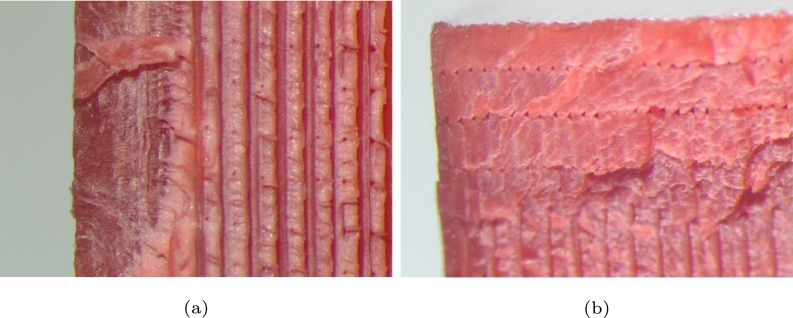
Internal failures due to the square waveform; (a) component failure at 23,150 cycles and (b) component failure at 27,236 cycles.

In this study is also employed an optical microscope to analyze the waveform effect on the internal layer of the failure, it was performed among samples with different load waves, as shown in Figure 8, Figure 9, Figure 10.

In Fig. 8a is clearly illustrated the crack growth, where the yellow circle indicates its initial position and direction of growth. It can also be seen where the start crack is a zone brittle, and after zone is a comport as ductile. Fig. 8b shows the two effects of fatigue: in the top part, the chevron pattern crack is present, and on the left is the striation generated by the failure propagation. Layers in 3d-printed components increase the stresses, due to the voids generated by the printing process can work as stress concentrators [28].

Fig. 9a presents the effect of fatigue on the failure of the material, the rubbed surface (a), the region of the beach marks and the effect of cycling in the bottom part (b), it can be an indicator of some resistance to fatigue. Fig. 9b displays the beach marks in the left zone. On the left bank, one can observe two shades; the reddest shade is an indicator of a fragile zone and rapid or sudden propagation, and the lighter shaded zone indicates ductility.

In Fig. 10a is shown the failure resulting from a square wave load. According to this figure, one can obtain a fan-shaped chevron pattern, which appears to have been encapsulated, and later, the fault is the product of it, only slower. It can also be seen that the direction of crack growth is preserved. Fig. 10b reports the crack growth and direction, and one can also see that the ductility of the sample is lower than previous samples. It can be an indicator of the duration in cycles is very low, even though the same load was used.

What emerges from the results reported here is that the effects from different waveform can directly influence the behaviours or types of failure in terms of brittleness or ductility are obtained, which increase or decrease accordingly. In the case of the sinusoidal and triangular waves, one can notice that they behave very similarly, and even the cycles that they support are almost identical. In the case of the square wave, the probe behaves as fragile, which is evident in the images and corroborates with the number of cycles. Regarding the experimental evidence on scatter, the major value is with a square wave because it is more aggressive than other waveforms. However, the average among the waveforms is between 0.1 and 0.2, and all the results are below the limit of 0.3 for the uniaxial test, as it is shown in Table 3.

## 6 Conclusions

This study set out to analyze the waveform load effects to improve the fatigue life prediction in 3D-printed PLA components. The second major finding was to predict its durability under real load conditions to control the accumulated damage using the fatigue strength curve.

The SN curve has identified similar results between the triangular and sinusoidal waveforms. Major scatter is found at intermediate load values between 8 and 14 MPa. The waveform effect is not as pronounced at low load values, however with values higher than 30% of the UTS an significant effect is visibly noted. The waveform has also an effect related to the dispersion of results; this can be seen at the average dispersion with a square waveform, which is the smallest one. The transition or reversal in the direction of the load also has an effect, in the case of the triangular wave is where a greater dispersion is observed, with a mean value 92.4% greater than the square. In the case of the sine wave, the mean value is 53.9% higher than the mean value of the square waveform dispersion.

The generalizability of these results is subject to certain limitations. For instance, all the tests are evaluated between 6 and 20 MPa, as can be seen in Table 2. Values outside this range need to be evaluated experimentally because of the dispersion found between load levels, and it is not possible to extrapolate the prediction of durability.

Notwithstanding these limitations, the study suggests that the load waveform can be used to establish a factor to translate the SN curve for a variable time history. The waveform has a significant effect related to the frequency of charge, and it is important to know the variability of the charge to develop a severity factor. This will improve the operational life prediction of components made of PLA. Further research should be carried out to establish the irregularity factor in complex time histories or with spectral processes.

*Funding*

The authors did not receive support from any organization for the submitted work.

## CRediT authorship contribution statement

Moises Jimenez-Martinez: Conceived and designed the experiments; Analyzed and interpreted the data; Contributed reagents, materials, analysis tools or data; Wrote the paper.

Julio Varela-Soriano: Performed the experiments; Analyzed and interpreted the data; Contributed reagents, materials, analysis tools or data.

José Jorge Rojas Carreón: Performed the experiments; Contributed reagents, materials, analysis tools or data.

Sergio G. Torres-Cedillo: Contributed reagents, materials, analysis tools or data; Wrote the paper.

## Declaration of Competing Interest

The authors declare that they have no known competing financial interests or personal relationships that could have appeared to influence the work reported in this paper.

## Data Availability

Data included in article/supp.material/referenced in article.
